# Effect of nanosecond pulsed electric fields (nsPEFs) on coronavirus survival

**DOI:** 10.1186/s13568-023-01601-3

**Published:** 2023-09-09

**Authors:** Jody C. Cantu, Ronald A. Barnes, Bryan M. Gamboa, Allen S. Keister, Ibtissam Echchgadda, Bennett L. Ibey

**Affiliations:** 1 General Dynamics Information Technology, JBSA Fort Sam Houston, San Antonio, TX USA; 2 Air Force Research Laboratory, 711Th Human Performance Wing, Airman Systems Directorate, Bioeffects Division, Radio Frequency Bioeffects Branch, JBSA Fort Sam Houston, San Antonio, TX USA; 3 Air Force Office of Scientific Research, Air Force Research Laboratory, Arlington, VA USA

**Keywords:** Coronavirus, Decontamination, Neutralization, Nanosecond electric pulses

## Abstract

Previous work demonstrated inactivation of influenza virus by GHz frequency electromagnetic fields. Despite theoretical and experimental results, the underlying mechanism driving this inactivation remains unknown. One hypothesis is that the electromagnetic field is causing damage to the virion membrane (and therefore changing spike protein orientation) rendering the virus unable to attach and infect host cells. Towards examining this hypothesis, our group employed nanosecond pulsed electric fields (nsPEFs) as a surrogate to radiofrequency (RF) exposure to enable exploration of dose response thresholds of electric field-induced viral membrane damage. In summary, Bovine coronavirus (BCoV) was exposed, in suspension, to mono and bipolar 600-ns pulsed electric fields (nsPEFs) at two amplitudes (12.5 and 25 kV/cm) and pulse numbers [0 (sham), 1, 5, 10, 100, and 1000] at a 1 Hz (Hz) repetition rate. The temperature rise immediately after exposure(s) was measured using thermocouples to differentiate effects of the electric field (E-field) and heating (i.e., the thermal gradient). Inactivation of BCoV was evaluated by infecting HRT-18G host cells and assessing differences in virus infectivity days after exposure. Our results show that 600 nsPEFs, both bipolar and monopolar, can reduce the infectivity of coronaviruses at various amplitudes, pulse numbers, and pulse polarity. Interestingly, we observed that bipolar exposures appeared to be more efficient at lower exposure intensities than monopolar pulses. Future work should focus on experiments to identify the mechanism underlying nsPEF-induced viral inactivation.

## Introduction

The response to the COVID-19 pandemic revealed a need for physical sterilization techniques, including technologies safe around humans that can be used in occupied public spaces (Martins et al. 2022). A promising technology involves using electromagnetic (EM) fields to neutralize microbes at intensities within the Institute of Electrical and Electronics Engineers (IEEE) safety standards (Yang et al. 2015). In support of this, recent studies suggest that EM waves can elicit a resonant effect on viruses, resulting in their inactivation (Gulyaev et al. 2020; Sun et al. 2017; Yang et al. 2015). Specifically, the exposure of microbes of sizes < 1 µm in diameter, which includes coronaviruses, to a high amplitude EM pulse can induce complex mechanical and physical breakdown of the material, including complete destruction of the virus (Epstein and Cook 1951; Gulyaev et al. 2020; Martens et al. 2020).

Our laboratory and others use nanosecond duration pulsed electric fields (nsPEFs) as a surrogate for the delivery of free-field radiofrequency (RF) pulsed exposures to evaluate the impact of high peak electric fields. Exposure to high-power, short duration electric pulses, such as nsPEFs, has been shown to cause permeabilization of cellular membranes (Gulyaev et al. 2020; Schoenbach et al. 2007). Specifically, nsPEF cellular studies showed that exposures can injure cell membranes by inducing permeabilization, which can result in the free passage of molecules across the plasma membrane (Cantu et al. 2016; Ibey et al. 2010; Pakhomov et al. 2007a; Pakhomov et al. 2007b; Schoenbach et al. 2007; Semenov et al. 2013; Vernier et al. 2006. Based on these studies showing an impact of nsPEFs on biological membranes, we hypothesized that similar exposures may be capable of impacting viral membranes. This hypothesis agrees with several recent reports suggesting that nsPEF-driven inactivation of virus should be investigated (Farmani et al. 2021; Ruiz-Fernández et al. 2022). Coronaviruses, in particular, are composed of a single-stranded ribonucleic acid (RNA) core surrounded by a lipid envelope with embedded viral glycoproteins (nucleocapsid, spike, envelope, haemagglutinin-esterase, and integral membrane proteins) (Clark 1993). Dissolution of the viral envelope renders it neutralized; therefore, some sterilization techniques could target the viral envelope to induce its breakdown (Lin et al. 2020). The lipid envelope is partially derived from portions of the host cell membrane (phospholipids) and, therefore, could be susceptible to the same nsPEF-derived forces that permeabilize biological lipid membranes.

In this study, we evaluated the effect of nsPEF (600 ns in duration) on the infectivity of bovine coronavirus (BCoV), a surrogate for the severe acute respiratory syndrome coronavirus 2 (SARS-CoV-2) virus. Specifically, we evaluated the effects of increasing the applied amplitude (12.5 vs 25 kV/cm) and pulse number (0–1000) to establish a range of inactivation thresholds for both monopolar (MP) and bipolar (BP) exposures. Furthermore, nsPEF-induced changes in viral titer were compared to temperature changes during exposure to distinguish if the effects on virus neutralization were related to temperature. Our results suggest that, while increased temperature during nsPEF exposure may play a role in the neutralization of BCoV, the decrease in infectivity following nsPEF exposure is more closely linked to energy deposition in the sample.

## Materials and methods

### HRT-18G cell culture and bovine coronavirus propagation

*Homo sapiens* ileocecal colorectal adenocarcinoma cells (HRT-18G, ATCC No. CRL-11663), were obtained from American Type Culture Collection (ATCC) (Manassas, Virginia). HRT-18G cells were maintained in Dulbecco’s Modified Eagle’s Medium (DMEM) supplemented with 5% fetal bovine serum (FBS) and 100 U/ml penicillin/streptomycin at 37 °C with 5% CO_2_ in air. The media and its components were purchased from ATCC (Manassas, Virginia).

Bovine coronavirus (BCoV) NR-445, Mebus was acquired from BEI Resources (Manassas, Virginia). All experiments containing BCoV were conducted in Eagle’s Minimum Essential Medium (EMEM, ATCC) supplemented with 100 U/ml penicillin/streptomycin. BCoV was propagated to create a virus stock via passage in host (HRT-18G) cells. In brief, HRT-18G cells were grown to confluent monolayers in 6-well plates. Prior to infection, cells were rinsed twice with serum free media (SFM) and inoculated with 200 µL of BCoV BEI stock. Virus-inoculated cells were incubated for 2 h at 37 °C, 5% CO_2_, 95% RH, then 1.8 ml of EMEM containing 2% FBS was added to each well. The cells were then incubated for 6 days (37 °C, 5% CO_2_, 95% RH) to allow infection, as evidenced by the appearance of cytopathic effects (CPE). This process was repeated twice with reinfection in successively larger cell culture vessels to create a large volume of BCoV stock for this study. BCoV stock was aliquoted in small volumes and frozen at − 80 °C until use.

### Bovine coronavirus exposure

**Fig. 1:**
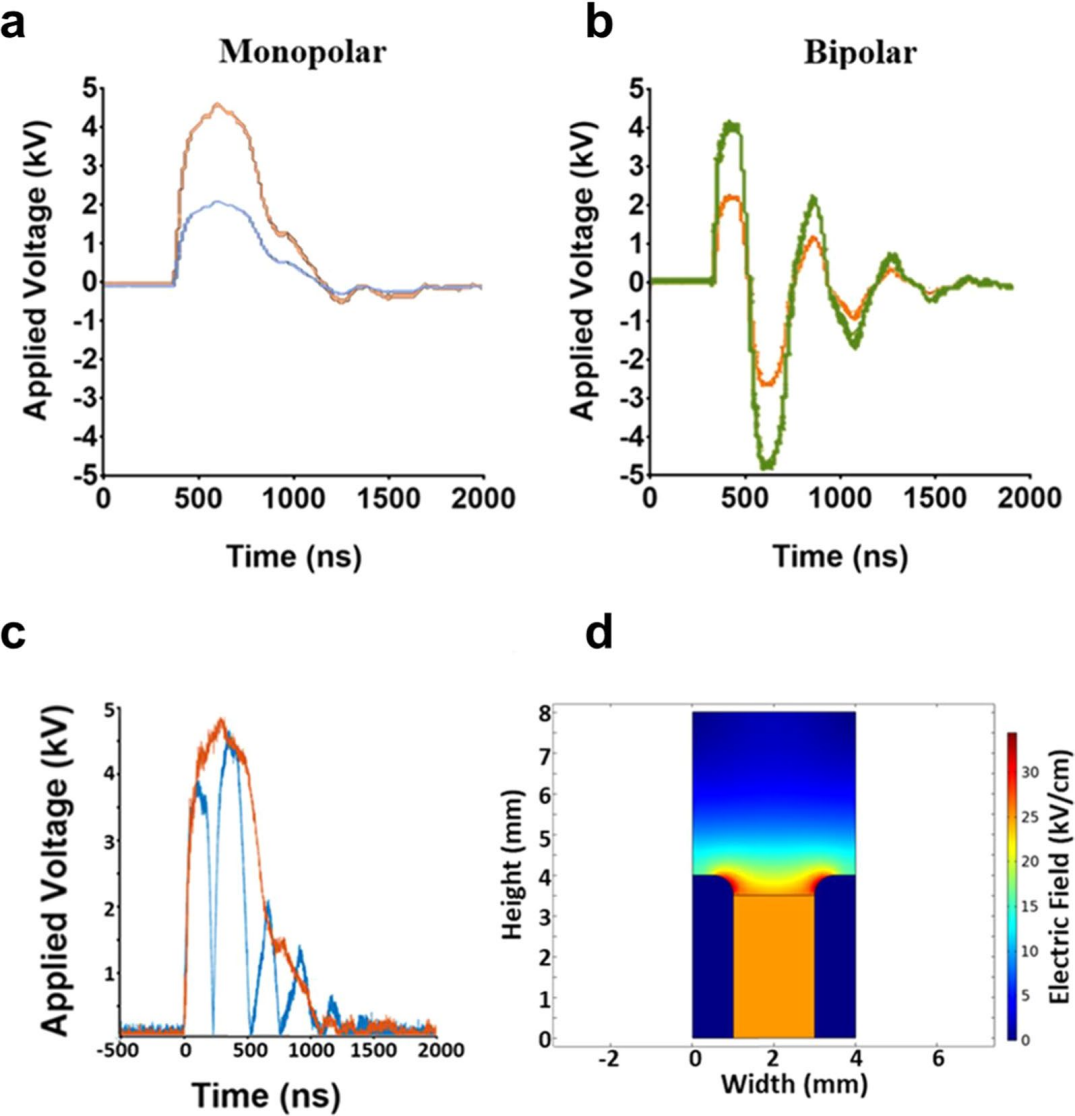
Monopolar (MP) and bipolar (BP) nsPEF exposure characterization. **a** Representative oscilloscope traces of the applied voltage used to deliver an E-field amplitude of 12.5 kV/cm MP (orange) or 25 kV/cm MP (blue) pulses. **b** Representative oscilloscope traces of the applied voltage used to deliver an E-field amplitude of 12.5 kV/cm BP (green) or 25 kV/cm BP (red) pulses. **c** Superimposition of MP and BP pulses. **d** COMSOL Multiphysics® software was used to model the predicted E-field distribution throughout the electroporation cuvette; the E-Field is uniform throughout the cuvette media

BCoV was exposed to MP (600 ns) or BP (300 + 300 ns) pulses as described previously (Ibey et al. 2014). Briefly, 450 µL of BCoV in solution was added to a conventional electroporation cuvette with a 2-mm gap. 600 nsPEF exposures were performed for 0 to 1000 pulses at an amplitude of 12.5 or 25 kV/cm and a 1 Hz repetition rate. Two Marx bank capacitor systems were used to generate either the 600 ns MP or 300 + 300 ns BP pulse. A high voltage power supply was used to charge the Marx bank. Delivery to the cuvette was achieved by a spark gap switch that discharged over an air gap between two conductive plates completing the circuit. The rate of discharge and amplitude was set by adjusting the charging voltage and the distance between the plates, respectively. The pulse delivered to the cuvette was measured using a high voltage probe connected to a high-speed oscilloscope (TDS3052B, Tektronix^®^, Beaverton, OR). Removal of the charging voltage controlled the number of pulses delivered, which were counted manually. The resultant pulse shapes are shown in Fig. 1a (MP) and Fig. 1b (BP). Superimposition of the 25 kv/cm MP and BP pulses is shown in Fig. 1c. For all exposures, we matched the peak of the highest amplitude BP component to the peak amplitude of the MP pulse (Fig. 1c).

For both exposure systems, COMSOL Multiphysics^®^ software v. 5.4 (COMSOL) was used to model electrodynamics and temperature within the exposure cuvette. The predicted E-field spatial distribution displays uniformity throughout the exposure solution (Fig. 1d) and, therefore, the E-field is assumed to be spatially uniform throughout the BCoV exposures.

### Evaluation of temperature in exposure system

**Fig. 2:**
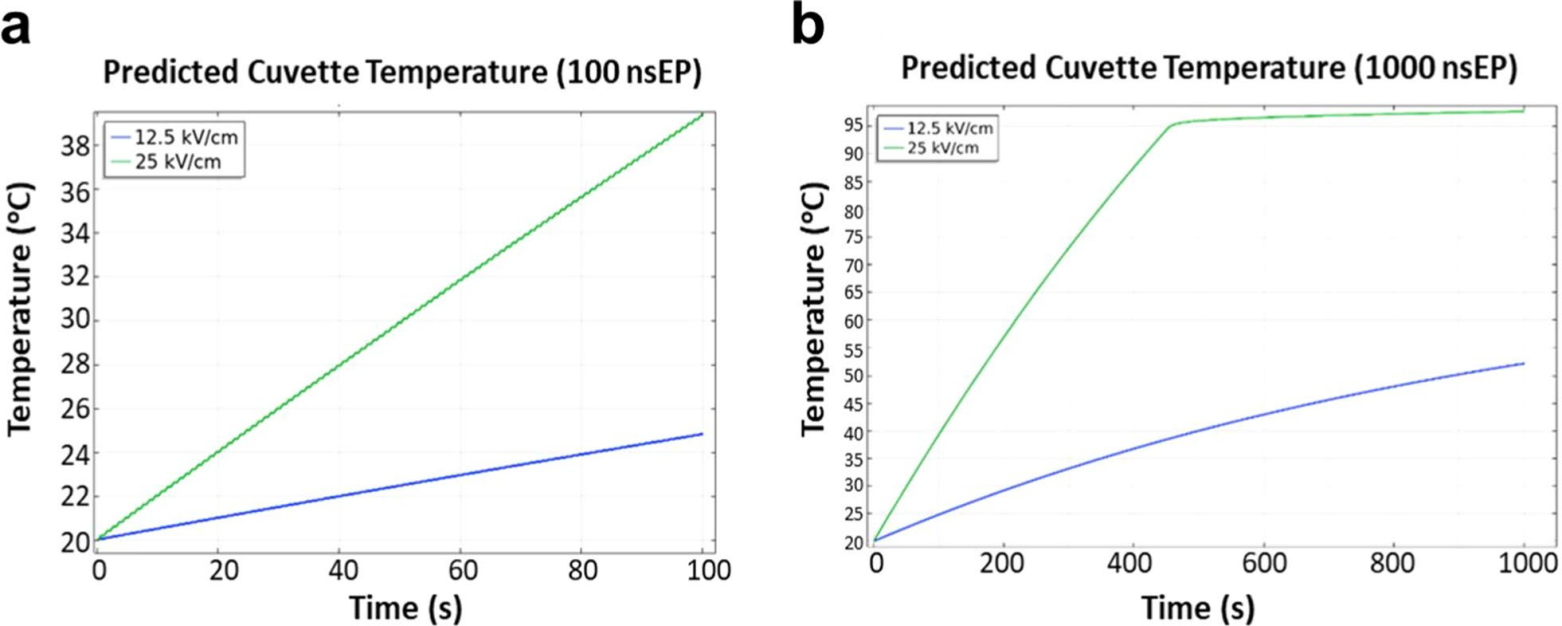
COMSOL cuvette thermal model results for 25 kV/cm and 12.5 kV/cm MP pulses. **a** 100 Pulses; **b** 1000 Pulses

Temperature rise during the exposure was predicted using COMSOL Multiphysics modeling software. Phase change in the media (i.e., boiling) is expected after approximately 500 pulses as seen in the model in Fig. 2. A solution conductivity of approximately 1.3 S/m and a contact resistance between the aluminum cuvette electrode plates/solution of 8 ohms was assumed. The solution reaches near boiling temperatures of approximately 100 °C, limiting the continued rise in temperature. Pulse to pulse cooling increases as temperature rises because of the heat capacity of the solution. To validate the model predictions an Opsens probe (OpSens, Inc., Quebec, Canada) was used to measure temperature within the cuvette. Prior to exposure, initial temperature of the BCoV solution (in the electroporation cuvette) was measured and recorded. After the specified exposure duration, the cuvette was quickly removed from the system, the cap removed, and an Opsens probe was inserted to evaluate the temperature rise following exposure.

### Evaluation of BCoV infectivity

**Fig. 3:**
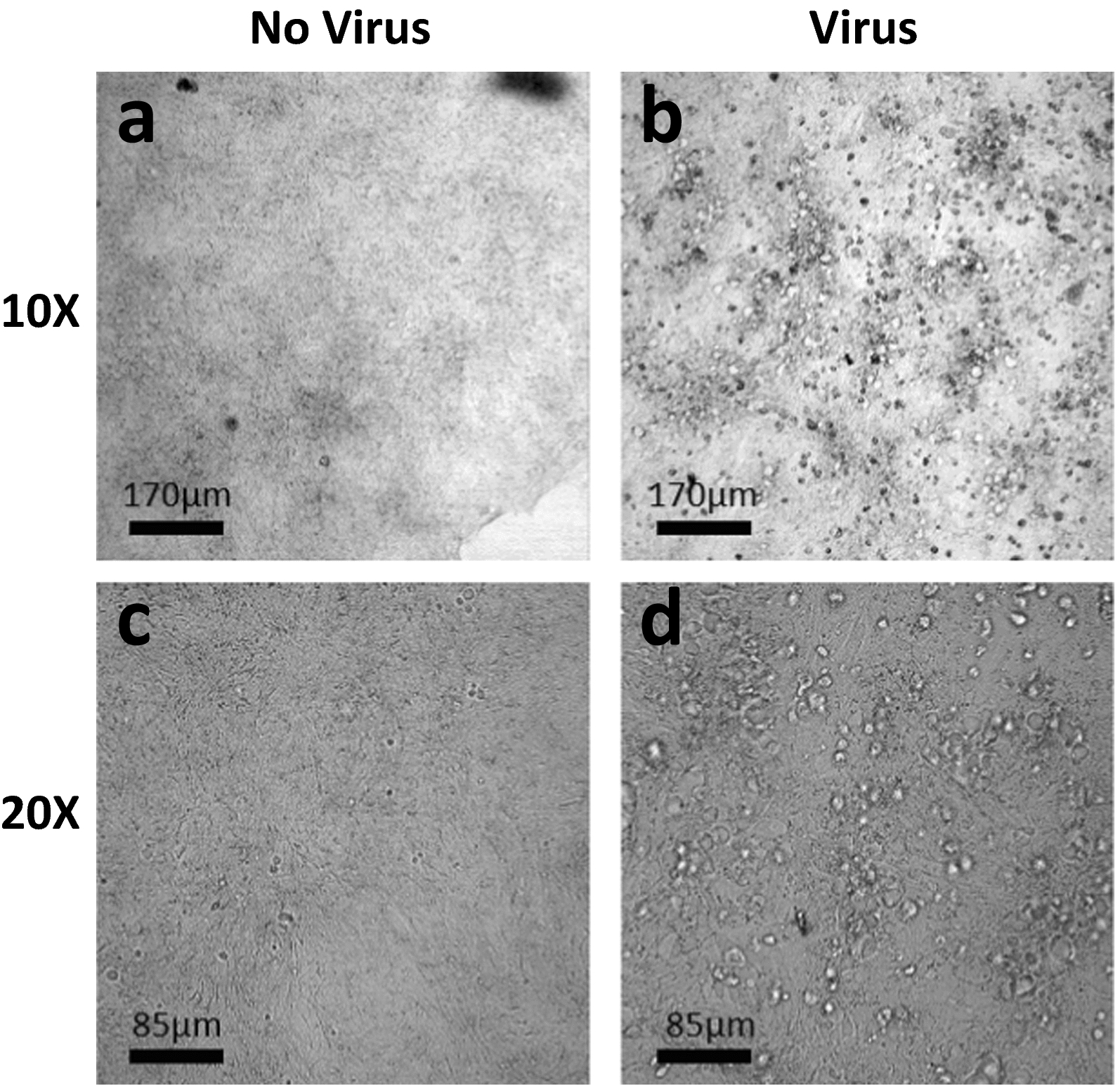
Representative microscopic images of CPE in HRT-18G host cells. **a**, **c** control cells (i.e. not infected) at 10 × (**a**) and 20 × (**c**) magnification. **b**, **d** virus-infected HRT-18G cells at 10 × (**b**) and 20 × (**d**) magnification showing CPE

After nsPEF exposure, each BCoV sample was diluted from 10^0^ to 10^–4^ in SFM and added to HRT-18G cells (plated at 15,000 cells per well in 96 well plates 24 h before) for virus titer determination to obtain the 50% tissue culture infectious doses (TCID_50_). Prior to infection, cell monolayers were rinsed twice with SFM, and then inoculated with 20 µL of diluted BCoV samples. Virus-inoculated cells were incubated for 1 h at 37 °C, 5% CO_2_, 95% RH, then 80 µL of EMEM containing 3% FBS was added to each well. The cells were then incubated for 6 days (37 °C, 5% CO_2_, 95% RH) to allow infection to occur. On Day 6 post-infection, three independent researchers evaluated each well microscopically to determine if CPE was present. Figure 3 shows the appearance of cells infected with virus at day 6 (Virus) in which CPE is evident vs non-infected cells (No Virus) that were incubated with media (control) showing a normal appearance. Researchers were blinded to the identity of the samples and results were evaluated as binary (i.e., infected or not infected). After evaluation, the titer of infectious virus was quantified by calculating the TCID_50_/ml for each condition. TCID_50_/ml was calculated using the Reed-Muench equation (Reed and Muench 1938). To compare results between multiple experiments (where definite TCID_50_/ml values may differ), we converted the TCID_50_/ml values to % Inactivation (ratio), as follows:

1 $$\%\, \mathrm{Infectivity}=\frac{\mathrm{TCID}50/\mathrm{ml\, Sample }\,(\mathrm{CPE})}{\mathrm{TCID}50/\mathrm{ml\, Sham }\,\left(\mathrm{CPE}\right)}\mathrm{x }100$$

2 $$\% \,\mathrm{Inactivation }\,\left(\mathrm{Ratio}\right)=100-\mathrm{\%\, Infectivity}$$

### Modeling of physical forces

To better evaluate the potential hypotheses that could explain the noted differences between MP and BP exposure, we estimated the mechanical forces acting on the viral particle in solution induced by a similar, analytically defined, electric field. For the membrane charging dynamics a single shell capacitive model and general parameters (membrane permittivity and conductivity, inside permittivity and conductivity) were taken from Kotnik and Miklavčič (2006). To describe membrane potential, we decomposed our model electric field pulse into frequency components and independently solved each frequency component of our model electric pulse. The net charging solution was reconstructed through simple superposition of each component. For brevity, we did not include a lengthy membrane charging model description.

The virion equations of motion were numerically solved with Mathematica (Mathematica, Princeton, New Jersey) using Stokes’ law, i.e., assuming the virion displacement was small, and the motion was sufficiently smooth. A virion is assumed to have a net charge on the order of 5 × 10^–15^ Coulombs for a naïve estimate based on the length of negatively charged RNA contained in a virion, 1 elementary charge per base-pair for 30 kilo-base-pairs (Cao et al. 2021) and mass of 1 fg (Bar-On et al. 2020). These assumptions allow for treatment of virion motion in one-dimension

$$\frac{dx\left(t\right)}{dt}=v\left(t\right), \frac{dv\left(t\right)}{dt}=q\frac{E\left(t\right)}{m}-\frac{\partial }{m} v(t),$$

With x(t): the virion position over time, v(t): the virion velocity, q: the virion charge, ∂: the drag coefficient of a 50 nm sphere (Turoňová et al. 2020), and m: the virion mass. With this methodology it is important to note that regardless of distinct virion parameters these equations remain valid, varying only in magnitude and direction based on actual virion net charge and mass.

### Statistics

All experiments were performed in triplicate and a mean and standard error of the mean (S.E.M.) were calculated. Error bars are provided as ± S.E.M. Pairwise comparisons of virus titers (TCID_50_/ml) or % infectivity for each nsPEF exposure were compared to control/sham (0 pulses) using pairwise Student’s t-test. The criterion for significance was set at p < 0.05 for a type I error.

## Results

### Thermal gradient following 600 nsPEF exposures

**Fig. 4:**
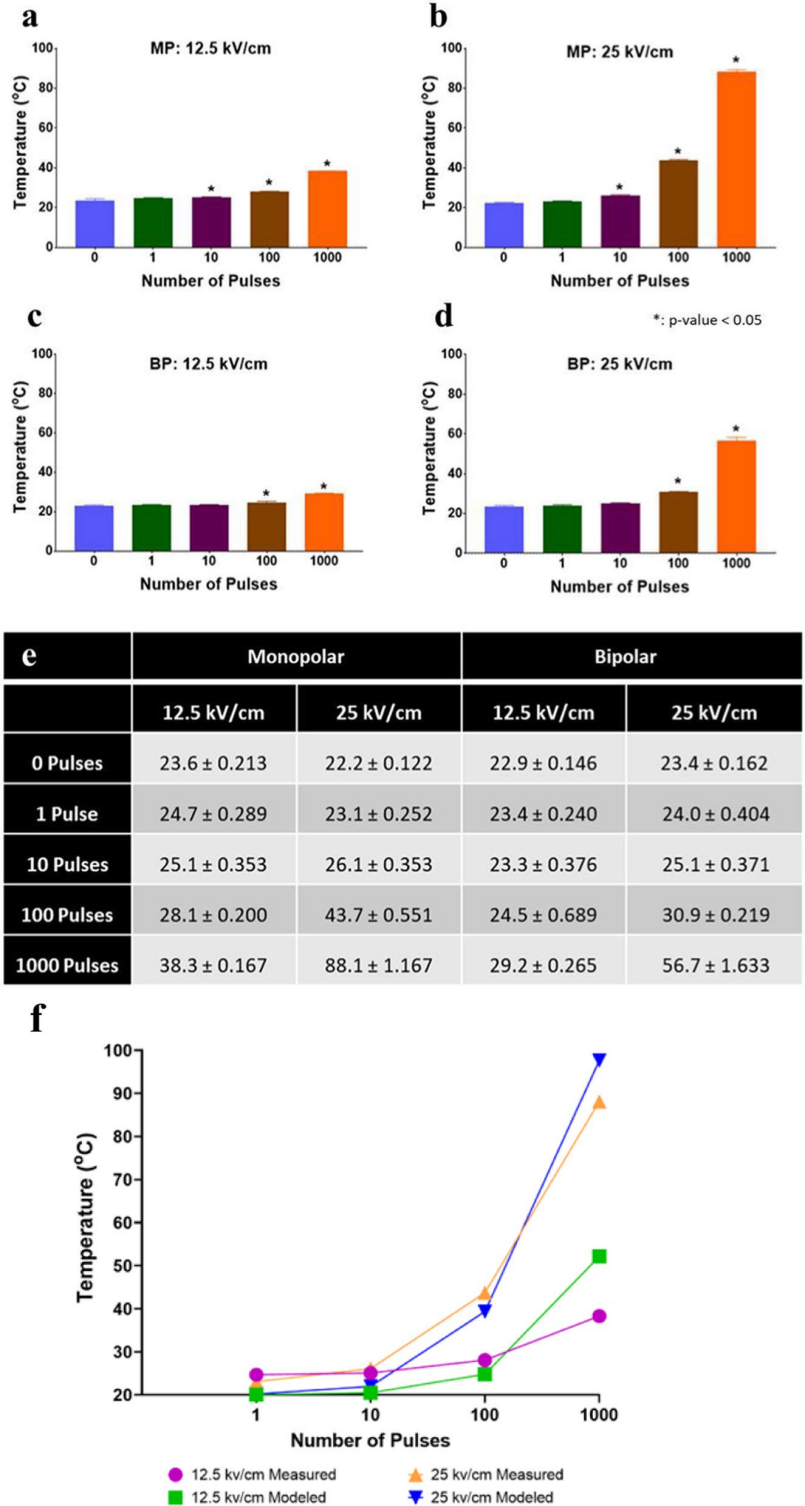
Temperature profiling of all nsPEF exposure conditions. **a**, **b** Final temperature (in °C) after MP pulsing at 12.5 kV/cm or 25 kV/cm, respectively. **c**, **d** Final temperature after BP pulsing at 12.5 kV/cm or 25 kV/cm, respectively. **e** All data shown in panels **a**–**d** are summarized in panel (**e**). Data are expressed as mean temperature values ± S.E.M. of at least three independent experiments (n = 3). Statistically significant differences are noted by an asterisk, which represents p < 0.05. Panel ** f **compares the measured temperature data (provided in panel** e** for the MP pulses) and COMSOL thermal modeled temperature data (described in Fig. 2)

The temperature of the BCoV solution (EMEM) was measured for each amplitude and pulse train used in this study (Fig. 4a–f). Results showed that increasing the pulse number increased the overall temperature within the sample, for each respective exposure. Additionally, increasing the amplitude of the pulses enhanced the thermal gradient during exposure (Fig. 4a vs b and c vs d). MP pulses produced greater overall temperature rises compared to their matched BP pulsed conditions (Fig. 4a vs c and b vs d). The rise in post-exposure measured temperature also corelates with that predicted by COMSOL modeling, as shown for MP pulses performed for the 12.5 kV/cm and 25 kV/cm amplitudes (Fig. 4f), further corroborating the results. Importantly, this difference in temperature between the two exposure paradigms was observed as we chose to match the peak electric field amplitude and duration of the exposures (Fig. 1C). Therefore, the total energy in each exposure is different due to the phase changes in the bipolar exposures resulting in a lower achieved temperature for bipolar exposures of the same pulse number and amplitude.

Importantly, previous studies have shown the minimum temperature required to efficiently inactivate coronavirus is 60 °C, and the heat must be sustained for prolonged periods of time (> 15 min) (Burton et al. 2021; Kampf et al. 2020). Accordingly, in our experiments, the only condition that generated heat capable of completely inactivating BCoV was 1000 MP pulses at an amplitude of 25 kV/cm.

### Inactivation of BCoV following MP 600 nsPEF exposure

**Fig. 5:**
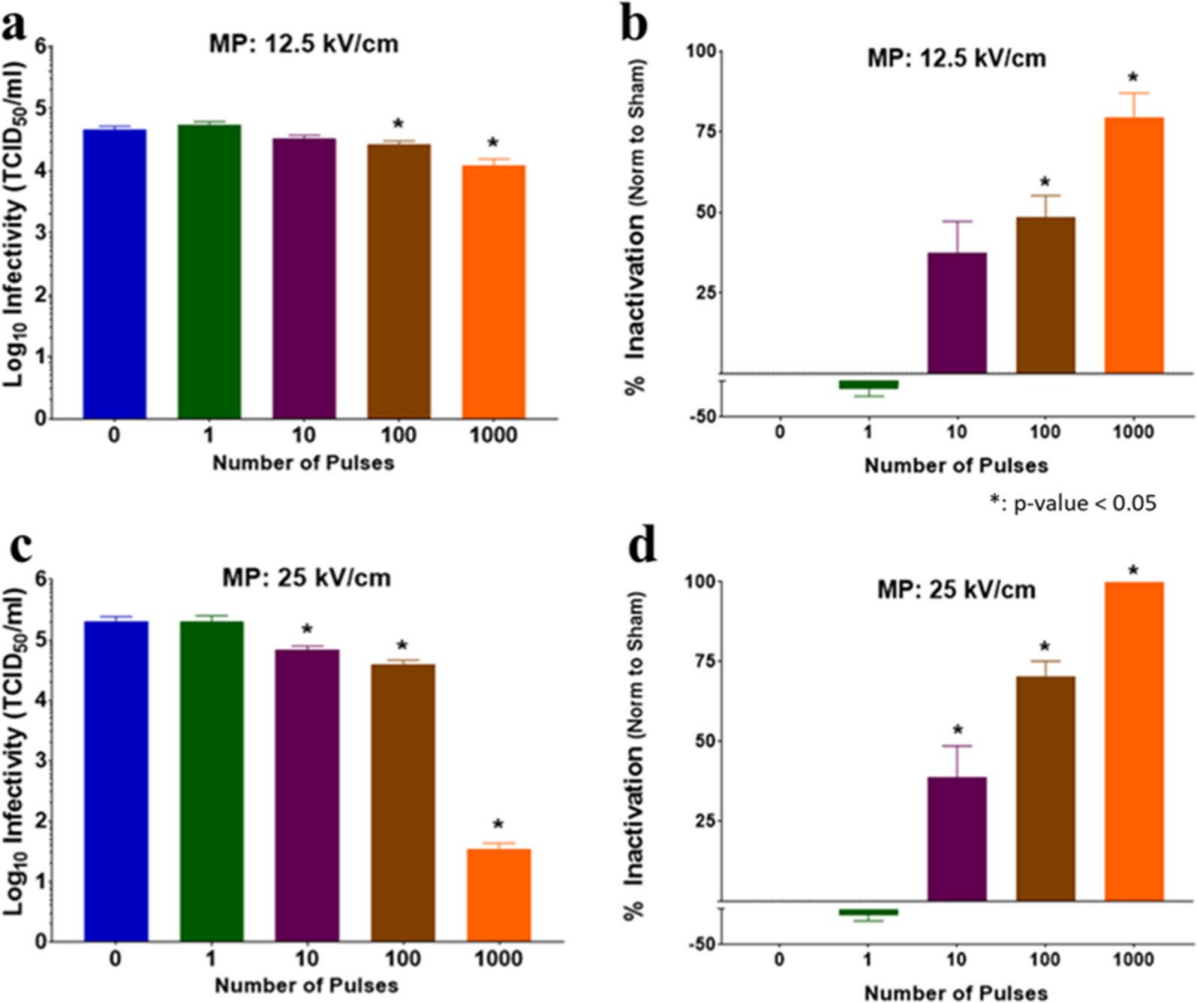
Infectivity of BCoV after exposure to MP nsPEFs for different pulse numbers and E-fields. Viral infectivity was assessed by CPE evaluation. Viral titers were determined upon titration on HRT-18G cells, as described in the methods, and evaluated at 6 days post inoculation. **a** and **b** are the TCID_50_/ml and % Inactivation (i.e., data normalized to non-exposed virus sham control/0 pulses) following exposure to 12.5 kV/cm MP pulses. **c** and **d** are the TCID_50_/ml and % Inactivation following exposure to 25 kV/cm MP pulses. Data are expressed as mean values ± S.E.M. of at least three independent experiments (n = 3). Statistically significant differences are noted by an asterisk, which represents p < 0.05

The inactivation of BCoV was evaluated by assessing viral titer (TCID_50_/ml) following exposure to 600 ns MP pulses (0, 1, 10, 100, or 1000) at two amplitudes (12.5 kV/cm or 25 kV/cm) based on the formation of CPE in cultured cells. For the 12.5 kV/cm, results show statistically significant BCoV inactivation starting at 100 pulses (Fig. 5a and b). In the higher amplitude field, inactivation of BCoV was significant following exposure to 10 pulses (Fig. 5c and d). In both conditions, the inactivation increased as amplitude and/or pulse number increased, with maximal “% inactivation” at 1000 pulses for both amplitudes.

### Inactivation of BCoV following BP 600 nsPEF exposure

**Fig. 6:**
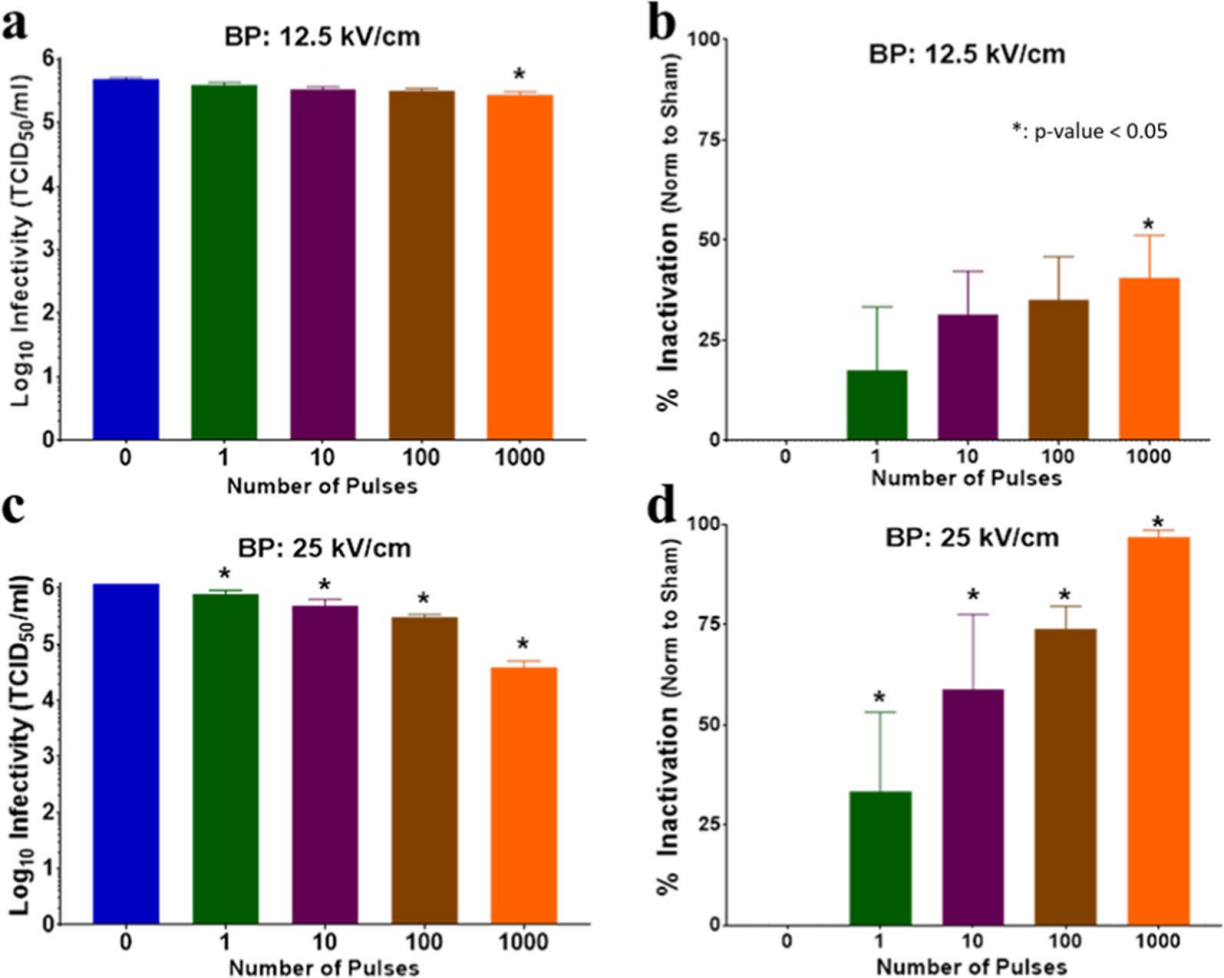
Infectivity of BCoV after exposure to BP nsPEFs for different pulse numbers and E-fields. Viral infectivity was assessed by CPE evaluation. Viral titers were determined upon titration on HRT-18G cells, as described in the methods, and evaluated at 6 days post inoculation. **a** and **b** are the TCID_50_/ml and % Inactivation (i.e., data normalized to non-exposed virus sham control/0 pulses) following exposure to 12.5 kV/cm BP pulses. **c** and **d** are the TCID_50_/ml and % Inactivation following exposure to 25 kV/cm BP pulses. Data are expressed as mean values ± S.E.M. of at least three independent experiments (n = 3). Statistically significant differences are noted by an asterisk, which represents p < 0.05

BP pulses, which are characterized by a reversal of polarity halfway through the pulse duration, have been shown to be as effective as MP exposures for micro and millisecond pulse durations. However, for nanosecond duration pulses, BP pulses are markedly less effective as compared to energy-matched MP pulses. Using second harmonic imaging, it was shown that BP nsPEF exposures impact both sides of a mammalian cell similarly to longer pulses but fail to induce as significant permeabilization. The mechanism behind “BP cancellation” remains unknown but has been repeatedly observed by several laboratories in different studies (Gianulis et al. 2018; Ibey et al. 2014; Moen et al. 2016; Pakhomov et al. 2018). As BP exposures match free-field exposures better, we exposed BCoV to BP (300 + 300 ns) pulses as a comparison to 600 ns MP pulses. As shown in Fig. 1 and Fig. 4, the overall energy within the BP pulse is ~ 30% less than the MP pulse. We chose to match peak electric field amplitude rather than energy, hypothesizing that peak electric field amplitude would be the driver of virus inactivation. Notably, the temperature profile for BP pulse exposure shows less of a temperature increase at both amplitudes tested in this study (Fig. 4c–d) as expected. We exposed BCoV to BP pulses (0, 1, 10, 100, or 1000) at two amplitudes (12.5 kV/cm or 25 kV/cm) and evaluated the infectivity of exposed virion based on the formation of CPE in cultured cells. Results shows statistically significant inactivation of BCoV only at the 1000 pulse condition at low (12.5 kV/cm) amplitude (Fig. 6a and b). However, we observe statistically significant inactivation of BCoV with as low as 1 pulse of 25 kV/cm nsPEF that increases with increasing pulse number (Fig. 6c and d).

### Relationship between BCoV inactivation and exposure properties

**Fig. 7:**
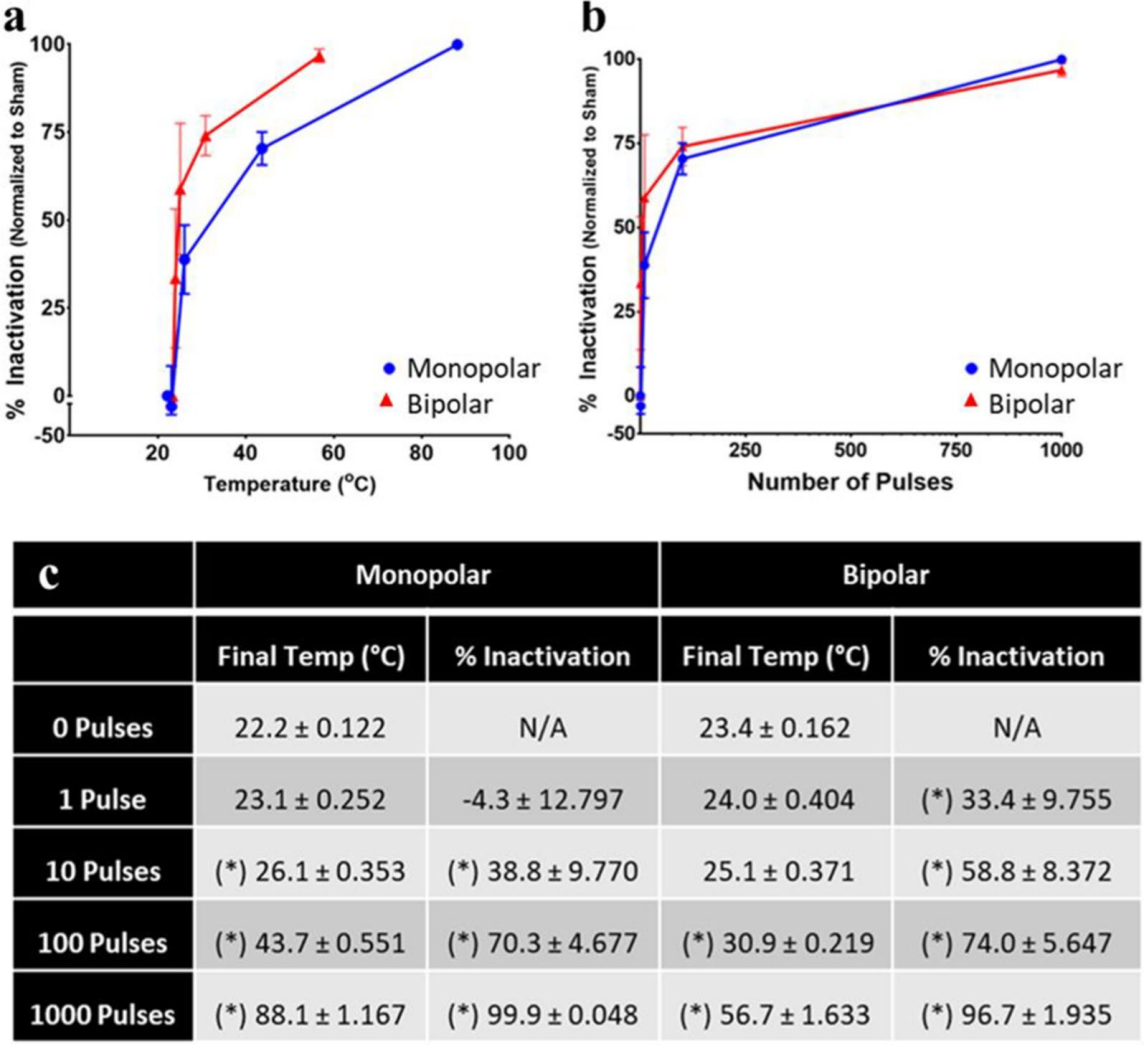
Percent inactivation in MP versus BP pulsing as a function of temperature and pulse number. Numerical data from the figures shown in panels **a** and **b** are summarized in panel (**c**). Data are expressed as mean values ± S.E.M. of at least three independent experiments (n = 3). In panel **c**, statistically significant differences are noted by an asterisk, which represents p < 0.05

To differentiate between the properties of nsPEF that induces inactivation, we plotted BCoV infectivity data (MP or BP pulses) at 25 kV/cm as a function of sample temperature (Fig. 7a) or pulse number (Fig. 7b). Results show that BP pulsing induces inactivation of BCoV at lower temperatures than MP pulsing equivalents (Fig. 7a and c).

### Modeling of membrane charging and mechanical forces

**Fig. 8:**
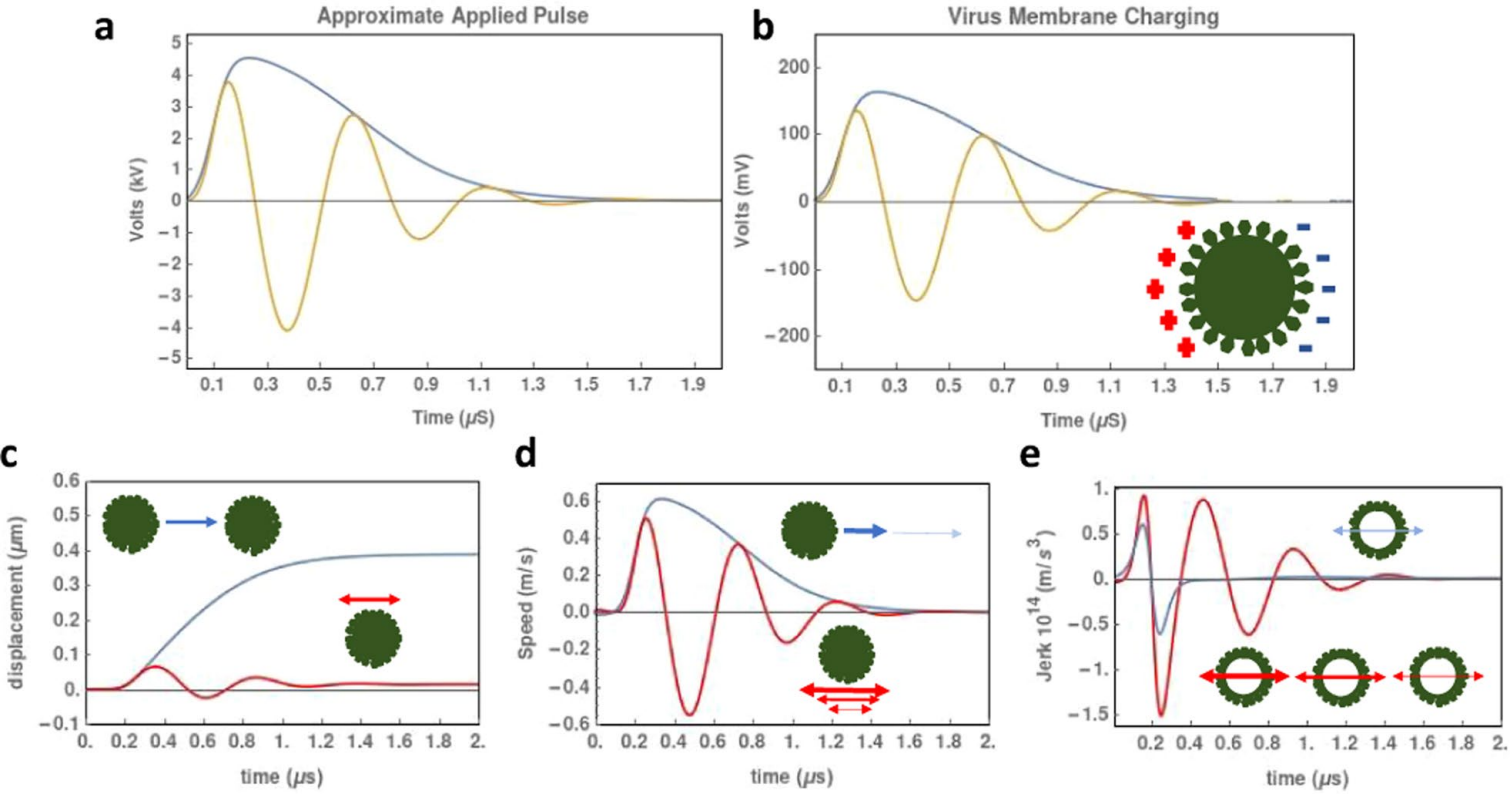
Modeling nsPEF charging on viral membrane and resultant mechanical forces. **a** The applied pulse shape used in the simulation to represent MP and BP exposures of BCoV. **b** The resultant membrane charging observed on the viral membrane (100 nm diameter). **c** Mechanical forces acting on the cell due to the nsPEF electric field: **c** displacement **d** Velocity and **e** Jerk

Results of the charging model is presented in Fig. 8. The applied voltage and the resultant charging on the viral membrane are presented in Fig. 8a and b. An inset cartoon of the viral membrane depicts the direction of charging on the viral membrane (which notable oscillates for bipolar exposures). The blue line and yellow line represent the unipolar and bipolar modeling results, respectively. The results of the mechanical models are presented in three panels with inset cartoons illustrating the direction of the physical forces on the viral membrane (Fig. 8c–e). The blue line and red line represent the unipolar and bipolar modeling results, respectively.

## Discussion

A major lesson from the COVID-19 pandemic is that physical sterilization methods that kill viruses but are not harmful to humans are required in order to minimize the spread and infectivity of microorganisms in occupied spaces (Martins et al. 2022). Both ultraviolet and chemical treatments have proven effective but can be unsafe in the presence of humans. A leading non-chemical technology involves EM fields (EMFs), which may be developed and used at a low intensity; bystander safe level, yet remain capable to inactivate microorganisms (Perlman et al. 2021; Sun and Liu 2011). In this paper, we investigated the ability of nsPEF exposures to neutralize virus infectivity as a direct application technology (liquid samples) and as a surrogate for high peak power pulsed electric fields (surface decontamination).

To date, the mechanism(s) and properties of viruses that would enable EM-based neutralization remain largely unknown. This void in knowledge necessitates more modeling and experiments to help determine if electrical pulses can be effective virus neutralizers. We conducted a comprehensive investigation exposing a surrogate coronavirus, BCoV, to various nsPEFs exposure conditions to precisely define the EM parameters that will neutralize the virus. Previous approaches to studying coronavirus neutralization used surrogate agents including influenza or bacteriophage MS2, due to the stringent requirement of biosafety level 3 (BSL3) laboratory associated with handling SARS-CoV-2. In our experiments, we selected a BCoV as a model virus for SARS-CoV-2 to better match the morphology and shape of SARS-CoV-2) as well as for its biosafety level (BSL2) requirement (Abdelrahman et al. 2020; Cantu et al. 2023; Echchgadda et al. 2023; Vlasova et al. 2021; Yao et al. 2020; Clark 1993). The two beta-coronaviruses are enveloped positive-sense single-stranded RNA viruses with similar size and composition (Cantu et al. 2023). We believe our selection of BCoV would provide a greater reliability in translating our experimental results to applicability in other coronaviruses neutralization.

Furthermore, to our knowledge, we are the first group to study the effect of nsPEFs on virus neutralization, particularly coronavirus inactivation. Specifically, we compared MP and BP nsPEF exposures of BCoV to define the dose needed to achieve viral neutralization. Results showed significant inactivation of BCoV even in the absence of a thermal rise (Fig. 7). Specifically, MP and BP pulses induce similar inactivation of B CoV at 100 pulses (70.3 ± 4.677% and 74.0 ± 5.647%, respectively) even though the temperature of the two solutions is significantly different (43.7 °C and 30.9 °C).

While we do not conclusively identify the specific mechanism by which nsPEF neutralizes BCoV in the present study, our results suggest the observed viral inactivation may involve multiple forces acting upon the virus particle. For decades, researchers have described the effects of electrical pulses on lipid bilayers using model membranes and MD simulations (Böckmann et al. 2008; Cantu et al. 2016; Eisenberg et al. 1973; Ibey et al. 2009; Joshi et al. 2001; Kramar et al. 2007; Tarek 2005; Teissie and Tsong 1981; Tieleman et al. 2003; Vernier et al. 2009; Vernier et al. 2006). NsPEF exposures are known to induce multiple physical forces including electrical, mechanical, and thermal when delivered in biologically relevant solutions (Barnes et al. 2017; Roth et al. 2015, 2017; Schoenbach et al. 2007). In the present study, we observe equivalent inactivation of BCoV under conditions where temperatures do not meet the minimum threshold of heat required to neutralize BCoV (Figs. 4 and 6). Therefore, it appears that nsPEF exposure does not neutralize BCoV solely through thermal mechanisms.

In Fig. 8, we provide an analytical model of membrane charging (b) during a simulated exposure (a) showing that the membrane of the viron, despite being two orders of magnitude smaller than a mammalian cell (100 nm vs. 10 μm), shows significant membrane charging during both MP and BP exposures. The membrane potentials reached during the exposures are below what is considered “permeabilizing” for mammalian cells (1 V). However, as thresholds for viral permeabilization are unknown as well as the impact of repetitive exposures, we cannot exclude direct permeabilization of the viron membrane as a mechanism. That being said, we would not expect BP stimulation to induce more inactivation than MP exposures (Fig. 7) based on previous cellular results showing a marked inefficiency of BP nsPEF-induced permeabilization (Ibey et al. 2014). Future work will focus on devising methods to directly visualize loss of membrane integrity in viral particles to verify if rapid permeabilization is indeed occurring.

Other mechanisms that could result in inactivation include electromechanical and thermoelastic interactions. Thermoelastic phenomena occur when a media experiences a rapid temperature rise in a short time (< 10 µs). These thermoelastic phenomena have been postulated to contribute to effects such as electroporation. Previous work has shown that nsPEF pulses, specifically in a cuvette are capable of generating acoustic shock waves, but those shock waves appeared weaker than forces that would be expected to permeabilize membranes based on the thresholds from ultrasound and sonoporation literature (Roth et al. 2014). Future work will focus on measuring these forces within the cuvette and applying equivalent acoustic waves to virus in media to evaluate whether inactivation occurs. Additionally, electromechanical interactions occur due to a charged membrane interacting with an external electric field. The resulting electromotive forces could play a role in membrane damage through compressive, expansive or shear stresses. In Fig. 8c–e, we evaluated the electromotive forces of the electric field on a viron sized particle. We found that displacement would occur in MP and BP pulses at the microsecond timescale. Given the rate of pulsing of 1 Hz, we do not expect displacement effects to compound with subsequent pulsing due to time for dissipation and random motion within the virus containing solution. The velocity is also greater in the MP exposures leading to a rapid increase in velocity followed by a gradual slowing of the particle in the microsecond time frame. BP pulses appear to have overall no net movement of the cell and no net velocity change. However, when we evaluate the jerk imposed on the cell, we see that BP exposures generate a significant push pull force on the viron particle that is diffused by motion and velocity in the MP exposure. This jerk force could be responsible for the observed difference between MP and BP stimulation as its intensity is amplified for BP exposures. Future work will focus on isolating electromechanical forces and studying their impact on viral infectivity.

Lastly, high strength electric fields applied to virus could directly impact the spike protein conformation and therefore limit binding to the host cell. Specifically, the binding of the SARS-CoV-2-spike protein to the ACE2 receptor on the host cell has been shown to be impacted by the presence of a strong electric field through molecular dynamics modeling (Arbeitman et al. 2021). In this paper, force fields up to 10^7^ V/m were applied to spike protein and notable changes were observed within 100 ns. Extremely high field intensities at 10^9^ V/m changes were shown to be irreversible. Another paper also showed that at field intensities of 10^8^ V/m spike protein conformation could be modified on the nanosecond timescale (Kuang et al. 2022). In both papers it was shown that spike proteins are more susceptible to electric fields than other proteins and therefore, technology taking advantage of this vulnerability could be pursued to mitigate the infectivity of virus in a variety of settings. In our research, we directly applied 2.5 MV/m and 1.25 MV/m fields to a solution of virus. These fields are notably lower than the peak fields simulated in the two publications, but fields as low as 10^4^ V/m were predicted to induce some confirmational changes. It is unclear how the viral environment within the cuvette exposure performed in our study mirrors a MD simulation as far as true e-field intensity at the spike protein itself and how such fields in an unconstrained environment would compare to artificially forced field alignment with an isolated spike protein. However, as the field intensities applied (on the lower end of the simulations) are similar and the exposure duration comparable, it is important to follow up our experimental efforts with subsequent molecular level modeling to close this comparison gap.

To gain insight into the mechanism of action for viral impact by nsPEF it is important that we consider not only the vulnerability of the virus, but also the multiple forces acting simultaneously in the exposure medium. While we supply an electric field between two electrodes below the breakdown threshold, there remains current generation, thermal buildup, and mechanical forces in the system which can have an impact on the viral particles themselves. As is true for previous nsPEF exposures in cells and tissues, we create multiple physical forces simultaneously in solutions when we discharge high voltage in a short time period. This creates multiple forces acting on the target of interest and it is unlikely that any one is entirely driving the biological response. Previous work has investigated this for cellular exposures with some conclusions related to membrane permeabilization of the plasma membrane, however, as the target has become small and has different physical vulnerably, such studies should be repeated to better understand the impact of each physical force within the exposure system. (Roth et al. 2015; Barnes et al. 2017**)**. Understanding which force dominates in achieving the desired biological impact is critical to refining a technological approach to viral decontamination. Therefore, we should consider the possibility of the field having a direct impact on the molecular components of the virus.

In conclusion, the present study shows the efficacy of 600 nsPEFs to inactivate coronavirus at various amplitudes, pulse numbers, and pulse polarity even under conditions that do not produce neutralizing heat. Thus, these results could form a foundation for defining specific EMF exposure conditions that can neutralize viruses. Furthermore, understanding these inactivation mechanism(s) may prove important to targeting a variety of micro-organisms (Guo et al. 2018; Haberkorn et al. 2021; Martens et al. 2020). Future studies should expand upon the results described herein to determine how nsPEF technologies can be utilized to safely disinfect virus-contaminated spaces for viral decontamination and to clarify the mechanism of interaction driving this inactivation.

## Data Availability

Data will be made available upon valid request.

## Abbreviations

ANOVA: Analysis of variance
ATCC: American type culture collection
BCoV: Bovine coronavirus
BP: Bipolar
CPE: Cytopathic effects
DMEM: Dulbecco’s modified eagle’s medium
E-field: Electric field
EM: Electromagnetic
EMEM: Eagle’s minimum essential medium
EMF: Electromagnetic field
FBS: Fetal bovine serum
Hz: Hertz
IEEE: Institute of Electrical and Electronics Engineers
MP: Monopolar
nsPEFs: Nanosecond pulsed electric fields
RF: Radiofrequency
RNA: Ribonucleic acid
SARS-CoV-2: Severe Acute Respiratory Syndrome Coronavirus 2
S.E.M.: Standard error of the mean
SFM: Serum free media
TCID_50_: 50% tissue culture infectious doses

## References

[CR1] Abdelrahman Z, Li M, Wang X (2020). Comparative review of SARS-CoV-2, SARS-CoV, MERS-CoV, and influenza A respiratory viruses. Front Immunol.

[CR2] Arbeitman CR, Rojas P, Ojeda-May P, Garcia ME (2021). The SARS-CoV-2 spike protein is vulnerable to moderate electric fields. Nat Commun.

[CR3] Barnes RA, Roth CC, Beier HT, Noojin G, Valdez C, Bixler J, Moen E, Shadaram M, Ibey BL (2017). Probe beam deflection optical imaging of thermal and mechanical phenomena resulting from nanosecond electric pulse (nsEP) exposure in-vitro. Opt Express.

[CR4] Bar-On YM, Flamholz A, Phillips R, Milo R (2020). SARS-CoV-2 (COVID-19) by the numbers. elife.

[CR5] Böckmann RA, de Groot BL, Kakorin S, Neumann E, Grubmüller H (2008). Kinetics, statistics, and energetics of lipid membrane electroporation studied by molecular dynamics simulations. Biophys J.

[CR6] Burton J, Love H, Richards K, Burton C, Summers S, Pitman J, Easterbrook L, Davies K, Spencer P, Killip M, Cane P, Bruce C, Roberts ADG (2021). The effect of heat-treatment on SARS-CoV-2 viability and detection. J Virol Methods.

[CR7] Cantu JC, Tarango M, Beier HT, Ibey BL (2016). The biological response of cells to nanosecond pulsed electric fields is dependent on plasma membrane cholesterol. Biochim Biophys Acta.

[CR8] Cantu JC, Butterworth JW, Mylacraine KS, Ibey BL, Gamboa BM, Johnson LR, Thomas RJ, Payne JA, Roach WP, Echchgadda I (2023). Evaluation of inactivation of bovine coronavirus by low-level radiofrequency irradiation. Sci Rep.

[CR9] Cao C, Cai Z, Xiao X, Rao J, Chen J, Hu N, Yang M, Xing X, Wang Y, Li M (2021). The architecture of the SARS-CoV-2 RNA genome inside virion. Nat Commun.

[CR10] Clark MA (1993). Bovine coronavirus. Br Vet J.

[CR11] Echchgadda I, Cantu JC, Butterworth J, Gamboa B, Barnes R, Freeman DA, Ruhr FA, Williams WC, Johnson LR, Payne J, Thomas RJA, Roach WP, Ibey BL (2023). Evaluation of viral inactivation on dry surface by high peak power microwave (HPPM) exposure. Bioelectromagnetics.

[CR12] Eisenberg M, Hall JE, Mead C (1973). The nature of the voltage-dependent conductance induced by alamethicin in black lipid membranes. J Membr Biol.

[CR13] Epstein M, Cook H (1951). The effects of microwaves on the Rous No. 1 fowl sarcoma virus. Br J Cancer.

[CR14] Farmani AR, Swanson RJ, Mahdavinezhad F, Shoormeij MH, Mohammadi S, Moeinzadeh A, Ghazipour F, Ai J (2021). Potential application of picosecond pulsed electric field (PPEF): advanced bioelectrical technology for potential COVID-19 treatment. J New Mater Electrochem Syst..

[CR15] Gianulis EC, Casciola M, Xiao S, Pakhomova ON, Pakhomov AG (2018). Electropermeabilization by uni-or bipolar nanosecond electric pulses: the impact of extracellular conductivity. Bioelectrochemistry.

[CR16] Gulyaev YV, Taranov IV, Cherepenin VA (2020). The use of high-power electromagnetic pulses on bacteria and viruses. Dokl Phys.

[CR17] Guo J, Dang J, Wang K, Zhang J, Fang J (2018). Effects of nanosecond pulsed electric fields (nsPEFs) on the human fungal pathogen *Candida albicans*: an in vitro study. J Phys D App Phys.

[CR18] Haberkorn I, Siegenthaler L, Buchmann L, Neutsch L, Mathys A (2021). Enhancing single-cell bioconversion efficiency by harnessing nanosecond pulsed electric field processing. Biotechnol Adv.

[CR19] Ibey BL, Xiao S, Schoenbach KH, Murphy MR, Pakhomov AG (2009). Plasma membrane permeabilization by 60-and 600-ns electric pulses is determined by the absorbed dose. Bioelectromagnetics.

[CR20] Ibey BL, Pakhomov AG, Gregory BW, Khorokhorina VA, Roth CC, Rassokhin MA, Bernhard JA, Wilmink GJ, Pakhomova ON (2010). Selective cytotoxicity of intense nanosecond-duration electric pulses in mammalian cells. Biochim Biophys Acta Gen Subj.

[CR21] Ibey BL, Ullery JC, Pakhomova ON, Roth CC, Semenov I, Beier HT, Tarango M, Xiao S, Schoenbach KH, Pakhomov AG (2014). Bipolar nanosecond electric pulses are less efficient at electropermeabilization and killing cells than monopolar pulses. Biochem Biophys Res Commun.

[CR22] Joshi RP, Hu Q, Aly R, Schoenbach KH, Hjalmarson HP (2001). Self-consistent simulations of electroporation dynamics in biological cells subjected to ultrashort electrical pulses. Phys Rev E.

[CR23] Kampf G, Voss A, Scheithauer S (2020). Inactivation of coronaviruses by heat. J Hospital Infect.

[CR24] Kotnik T, Miklavčič D (2006). Theoretical evaluation of voltage inducement on internal membranes of biological cells exposed to electric fields. Biophys J.

[CR25] Kramar P, Miklavcic D, Lebar AM (2007). Determination of the lipid bilayer breakdown voltage by means of linear rising signal. Bioelectrochemistry.

[CR26] Kuang Z, Luginsland J, Thomas RJ, Dennis PB, Kelley-Loughnane N, Roach WP, Naik RR (2022). Molecular dynamics simulations explore effects of electric field orientations on spike proteins of SARS-CoV-2 virions. Sci Rep.

[CR27] Lin Q, Lim JYC, Xue K, Yew PYM, Owh C, Chee PL, Loh XJ (2020). Sanitizing agents for virus inactivation and disinfection. View.

[CR28] Martens SL, Klein S, Barnes RA, TrejoSanchez P, Roth CC, Ibey BL (2020). 600-ns pulsed electric fields affect inactivation and antibiotic susceptibilities of *Escherichia coli* and *Lactobacillus acidophilus*. AMB Expr.

[CR29] Martins CV, Xavier CS, Cobrado L (2022). Disinfection methods against SARS-CoV-2: a systematic review. J Hosp Infect.

[CR30] Moen E, Ibey B, Beier H, Armani A (2016). Investigating membrane nanoporation induced by bipolar pulsed electric fields via second harmonic generation. Appl Phys Lett.

[CR31] Pakhomov AG, Kolb JF, White JA, Joshi RP, Xiao S, Schoenbach KH (2007). Long-lasting plasma membrane permeabilization in mammalian cells by nanosecond pulsed electric field (nsPEF). Bioelectromagnetics.

[CR32] Pakhomov AG, Shevin R, White JA, Kolb JF, Pakhomova ON, Joshi RP, Schoenbach KH (2007). Membrane permeabilization and cell damage by ultrashort electric field shocks. Arch Biochem Biophys.

[CR33] Pakhomov AG, Grigoryev S, Semenov I, Casciola M, Jiang C, Xiao S (2018). The second phase of bipolar, nanosecond-range electric pulses determines the electroporation efficiency. Bioelectrochemistry.

[CR34] Perlman SG, Forenza A, Heath RW, Saibi F, Cheponis M (2021) Systems and methods for electromagnetic virus inactivation. US Patent 17,224,977

[CR35] Reed LJ, Muench H (1938). A simple method of estimating fifty percent endpoints. Am J Epidemiol.

[CR36] Roth CC, Barnes RA, Ibey BL, Beier HT, Mimun LC, Maswadi SM, Shadaram M, Glickman RD (2015). Characterization of pressure transients generated by nanosecond electrical pulse (nsEP) exposure. Sci Rep.

[CR37] Roth CC, Glickman RD, Martens SL, Echchgadda I, Beier HT, Barnes RA, Ibey BL (2017). Adult human dermal fibroblasts exposed to nanosecond electrical pulses exhibit genetic biomarkers of mechanical stress. Biochem Biophys Rep.

[CR38] Roth CC, Maswadi S, Ibey BL, Beier HT, Glickman RD (2014) Characterization of acoustic shockwaves generated by exposure to nanosecond electrical pulses. In: Optical Interactions with Tissue and Cells XXV; and Terahertz for Biomedical Applications; SPIE 8941:262–271. 10.1117/12.2042184

[CR39] Ruiz-Fernández AR, Rosemblatt M, Perez-Acle T (2022). Nanosecond pulsed electric field (nsPEF) and vaccines: a novel technique for the inactivation of SARS-CoV-2 and other viruses?. Ann Med.

[CR40] Schoenbach KH, Hargrave SJ, Joshi RP, Kolb JF, Nuccitelli R, Osgood C, Pakhomov A, Stacey M, Swanson RJ, White JA (2007). Bioelectric effects of intense nanosecond pulses. IEEE Trans Dielectr Electr Insul.

[CR41] Semenov I, Xiao S, Pakhomova ON, Pakhomov AG (2013). Recruitment of the intracellular Ca2+ by ultrashort electric stimuli: the impact of pulse duration. Cell Calcium.

[CR42] Sun C-K, Tsai Y-C, Chen Y-JE, Liu T-M, Chen H-Y, Wang H-C, Lo C-F (2017). Resonant dipolar coupling of microwaves with confined acoustic vibrations in a rod-shaped virus. Sci Rep.

[CR43] Sun, C-K, Liu T-M (2011) Microwave resonant absorption method and device for viruses inactivation. US Patent 12,562,591

[CR44] Tarek M (2005). Membrane electroporation: a molecular dynamics simulation. Biophys J.

[CR45] Teissie J, Tsong TY (1981). Electric field induced transient pores in phospholipid bilayer vesicles. Biochem.

[CR46] Tieleman DP, Leontiadou H, Mark AE, Marrink S-J (2003). Simulation of pore formation in lipid bilayers by mechanical stress and electric fields. J Am Chem Soc.

[CR47] Turoňová B, Sikora M, Schürmann C, Hagen WJH, Welsch S, Blanc FEC, von Bülow S, Gecht M, Bagola K, Hörner C, van Zanbergen G, Landry J, de Azevedo NTD, Mosalaganti S, Schwarz A, Covino R, Mühlebach MD, Hummer G, Locker JK, Beck M (2020). In situ structural analysis of SARS-CoV-2 spike reveals flexibility mediated by three hinges. Science.

[CR48] Vernier PT, Sun Y, Gundersen MA (2006). Nanoelectropulse-driven membrane perturbation and small molecule permeabilization. BMC Cell Biol.

[CR49] Vernier PT, Levine ZA, Wu Y-H, Joubert V, Ziegler MJ, Mir LM, Tieleman DP (2009). Electroporating fields target oxidatively damaged areas in the cell membrane. PLoS ONE.

[CR50] Vlasova AN, Saif LJ (2021). Bovine coronavirus and the associated diseases. Front Vet Sci.

[CR51] Yang S-C, Lin H-C, Liu T-M, Lu J-T, Hung W-T, Huang Y-R, Tsai Y-C, Kao C-L, Chen S-Y, Sun C-K (2015). Efficient structure resonance energy transfer from microwaves to confined acoustic vibrations in viruses. Sci Rep.

[CR52] Yao H, Song Y, Chen Y, Wu N, Xu J, Sun C, Zhang J, Weng T, Zhang Z, Wu Z, Cheng L, Shi D, Lu X, Lei J, Crispin M, Shi Y, Li L, Li S (2020). Molecular architecture of the SARS-CoV-2 virus. Cell.

